# A SUMOylation-dependent HIF-1α/CLDN6 negative feedback mitigates hypoxia-induced breast cancer metastasis

**DOI:** 10.1186/s13046-020-01547-5

**Published:** 2020-02-24

**Authors:** Yiyang Jia, Yantong Guo, Qiu Jin, Huinan Qu, Da Qi, Peiye Song, Xiaoli Zhang, Xinqi Wang, Wenhong Xu, Yuan Dong, Yingying Liang, Chengshi Quan

**Affiliations:** The Key Laboratory of Pathobiology, Ministry of Education, College of Basic Medical Sciences, Jilin University, 126 Xinmin Avenue, Changchun, Jilin, 130021 People’s Republic of China

**Keywords:** Breast cancer, CLDN6, HIF-1α, SUMOylation, Tumor metastasis

## Abstract

**Background:**

We have previously described CLDN6 as a tumor suppressor gene in breast cancer. Here, a new finding is that CLDN6 was upregulated under hypoxia, a commonly recognized factor that promotes tumor metastasis. In this study, we aim to explain this confusing finding and delineate the role of CLDN6 in the breast cancer metastasis induced by hypoxia.

**Methods:**

RNAi and ChIP assays were used to confirm that CLDN6 is transcriptional regulated by HIF-1α. mRNA seq and KEGG analysis were performed to define the downstream pathways of CLDN6. The roles of the CLDN6/SENP1/HIF-1α signaling on tumor metastasis were evaluated by function experiments and clinical samples. Finally, the possible transcription factor of SENP1 was suspected and then validated by ChIP assay.

**Results:**

We demonstrated a previously unrecognized negative feedback loop exists between CLDN6 and HIF-1α. CLDN6 was transcriptionally up-regulated by HIF-1α under hypoxia. On the other hand, in cytoplasm CLDN6 combines and retains β-catenin, a transcription factor of SENP1, causing β-catenin degradation and preventing its nuclear translocation. This process reduced SENP1 expression and prevented the deSUMOylation of HIF-1α, ultimately leading to HIF-1α degradation and breast cancer metastasis suppression.

**Conclusions:**

Our data provide a molecular mechanistic insight indicating that CLDN6 loss may lead to elevated HIF-1α-driven breast cancer metastasis in a SUMOylation-dependent manner.

## Background

Intratumoural hypoxia is commonly found in breast cancer due to the rapid growth of tumour and abnormalities in the tumour vasculature, causing a significantly increased risk of breast cancer metastasis [1, 2]. The physiological response to hypoxia is mediated mainly by hypoxia-inducible factor 1 (HIF-1), a heterodimer composed of the oxygen-sensitive subunit HIF-1α and the stable subunit HIF-1β [3]. Under normoxic conditions, HIF-1α is hydroxylated by prolyl hydroxylase domain enzymes (PHDs) and then targeted by the ubiquitin ligase component von Hippel-Lindau (VHL) for degradation [4]. Hypoxia decreases the hydroxylation activity of PHDs and results in HIF-1α protein stabilization and translocation to the nucleus; here, HIF-1α dimerizes with HIF-1β and binds to hypoxia response elements (HREs; 5′-A/GCGTG-3′) in the genome, leading to the transcriptional activation of hundreds of genes including *VEGF*, *TWIST*, *SNAIL* and *GLUT*, thus promoting multiple steps within the metastatic cascade [5]. As such, HIF-1α degradation under hypoxic conditions is an essential homeostatic and tumour-suppressing mechanism.

Recent data indicate that HIF-1α stabilization is regulated not only by the conventional PHD-VHL system but also by other mechanisms [6–9]. SUMOylation, the conjugation of small ubiquitin-related modifier protein (SUMO) to a target protein, has been considered to play an essential regulatory role in HIF-1α protein stability [10–12]. SUMOylation of HIF-1α promotes recruitment of the modified protein to VHL through a PHD-independent mechanism, leading to ubiquitination and proteolysis even under hypoxic conditions. That is, SUMOylation serves as another direct signal of VHL binding for ubiquitin-dependent HIF-1α degradation [13]. SUMOylation is a dynamic process and can be reversed by sentrin/SUMO-specific proteases (SENPs) [14]. It has been reported that SENP1 can remove SUMO1 from SUMOylated HIF-1α and allows HIF-1α to escape degradation during hypoxia [13, 15].

Homeostatic signalling pathways often have built-in self-regulatory feedback mechanisms to attenuate their activation. HIF-1α is a major regulator that maintains oxygen homeostasis, and several feedback mechanisms involving HIF-1α have recently been revealed. It has been reported that a HIF-1α/LIMD1 negative feedback mechanism mitigates the pro-tumourigenic effects of hypoxia [16]. An HIF-1α target gene, NDRG3, reduced the hypoxic expression of HIF-1α by inhibiting AKT-driven translation of HIF-1α mRNA [17]. Another such gene, CITED2, acts as a negative feedback regulator and attenuates HIF-1α transcriptional activity by competing for TAZ1 binding [18].

Tight junctions (TJs) are composed of integral transmembrane and peripheral membrane proteins involved in complex protein-protein interactions [19]. CLDN6 is a 26-kDa TJ protein containing four transmembrane helices with a carboxyl-terminal tail extending into the cytoplasm [20]. The PDZ-domain-binding motif in the carboxy-terminal tail allows CLDN6 to interact with cytoplasmic TJ-associated proteins such as ZO1, β-catenin and cadherins thus regulating various signalling pathways [21]. We found that CLDN6 was transcriptionally upregulated by HIF-1α in three breast cancer cell lines. However, our recent work has shown that CLDN6 may be a tumour suppressor gene in breast cancer [22–24]. Little is known about the role of CLDN6 in cellular adaptation to hypoxia, whereas the roles of HIF-1α are well understood. Herein, a negative loop involving a SUMOylation-dependent feedback mechanism was identified to explain this seemingly contradictory result. In this study, we demonstrated that HIF-1α accumulation under hypoxia promotes CLDN6 transcription. On the other hand, increased CLDN6 weakens HIF-1α protein stability by reducing SENP1 expression and preventing the deSUMOylation of HIF-1α. This negative feedback loop contributes to oxygen homeostasis and slows down hypoxia-induced breast cancer metastasis.

## Materials and methods

### Cell culture

Human breast cancer cell lines MDA-MB-231, MCF-7, SkBr-3 and breast epithelial cell line HBL-100 were cultured in H-DMEM medium (Gibco, Carlsbad, CA, USA) containing 10% fetal bovine serum (HyClone Laboratories, Inc., Logan, UT USA) and 1% penicillin-streptomycin solution in a 5% CO_2_ humidified incubator at 37 °C. For hypoxic conditions, cells were incubated at 37 °C containing 1% O_2_, 5% CO_2_, and balance N_2_ in a humidified incubator.

### Plasmid and transfection

The pCMV-3 × FLAG-SENP1 plasmid and pCDNA3-HA-CTNNB1 plasmid were purchased from MiaolingBio (Wuhan, China). The CLDN6-GFP-luciferase overexpression lentivirus, CLDN6 RNAi lentivirus, HIF-1α RNAi lentivirus, CMV-3FLAG-HIF-1α ^WT^ plasmid and CMV-3FLAG-HIF-1α ^K391,477R^ plasmid were constructed by Genechem (Shanghai, China). CLDN6 shRNA target sequence is GGCAAGGTGTACGACTCA and HIF-1α shRNA target sequence is GTTACGTTCCTTCGATCAG. Lentiviruses or plasmids are transfected into cells according to instructions, and stable clones were generated as previously described [25].

### RT-PCR and real-time-RT-PCR

Total RNA was extracted from cells using TRIzol (Invitrogen) following the manufacturer’s instructions. One microgram of total RNA was subjected to reverse transcription using the One-Step cDNA Synthesis SuperMix (Transgene, Beijing, China). TransStart Green qPCR SuperMix (Transgene, Beijing, China) was used for real-time-RT-PCR. The PCR conditions and primer sequences are shown in Supplementary Material.

### Western blot assay

Cells were lysed with ice-cold RIPA lysis buffer containing phosphatase-protease inhibitor cocktails (Beyotime Biotechnology, Shanghai, China). The concentration of protein was measured by BCA Protein Assay Kit (Beyotime Biotechnology, Shanghai, China). Equal amounts of protein lysates were subjected to SDS gel electrophoresis, immunoblotted with primary antibodies, and then the matched secondary antibodies. Western blot results were quantified by using the Image J software. Antibodies used in this study were listed in Supplementary Material.

### RNA-Seq and KEGG pathway analysis

A Truseq RNA Sample Prep Kit (RS-122-2203, Illumina,San Diego, USA) was employed in cDNA library construction. The Illumina sequencing and foundation analysis were completed by Shanghai Majorbio Bio-pharm Biotechnology Co. (http://www.majorbio.com, Shanghai, China). KEGG analysis were conducted on all identified differentially expressed genes using the Goatools software.

### Wound healing assay

The cells were cultured in a 60 mm culture plate and were scratched using 200 μl pipette tips. Then the cells were rinsed using PBS and cultured in medium without FBS. Images were taken at 0 and 48 h to determine the width of the wounded area.

### Transwell invasion assay

Cell invasion experiment was performed using transwell (Corning, Lowell, MA, USA) containing 8.0 μm pore membranes covered with Matrigel (Corning, Lowell, MA, USA). The transwell was placed in a 24-well plate and cells were placed in the upper chamber of transwell. Subsequently, the cells were fixed in paraformaldehyde for 30 min and stained with crystal violet for 15 min. Finally, invasive cells on the lower surface of the membrane were counted by a microscope.

### Immunoprecipitation

Cells were lysed with ice-cold RIPA lysis buffer containing phosphatase-protease inhibitor cocktails (Beyotime Biotechnology, Shanghai, China). After centrifugation at 12000 rpm for 20 min, the protein supernatant was mixed with the specific primary antibody and incubated at 4 °C overnight. The protein-antibody complex was pulled down with Protein A/G PLUS-Agarose beads (Santa Cruz, CA, USA). After 4 h, those beads were collected and then boiled with SDS-PAGE buffer to release the binding protein, and the immunoprecipitated protein was eluted for western blot analysis.

### Chromatin immunoprecipitation assay

Cells were cross-linked with 4% formaldehyde, lysed with SDS buffer and sonicated. Sheared DNA was immunoprecipitated with specific primary antibody or normal mouse IgG (Proteintech, Shanghai, China) and pulled down by agarose beads as described above. The antibody/protein/DNA complex was washed according to manufacturer’s protocol (Cell Signaling Technology, MA, USA). DNA was extracted by phenol-chloroform and eluted for PCR. For the detection of HRE sequence, cells were cultured under hypoxia to obtain a considerable level of HIF-1α expression before treated with formaldehyde.

### Luciferase reporter assay

The promoter sequence of CLDN6 was inserted into pGL3 luciferase reporter and then transfected to MDA-MB-231 cells together with Renilla. Then cells were exposed to 1% O_2_ or transfected with PCMV-HIF-1α plasmid for 24 h and Dual-Luciferase® Reporter assay (Promega) was performed following manufacturer’s protocol.

### Subcellular fractionation

Isolation of nuclear and cytoplasmic extract was performed with a Nuclear Cytoplasmic Extraction Reagent kit (Transgene, Beijing, China) according to manufacturer’s protocol. For the detection of membrane bound proteins, the subcellular compartment proteins were isolated according to Baghirova’s protocol [26].

### Immunofluorescence

Cells were fixed with 4% paraformaldehyde for 10 min and then incubated with 0.1% Triton X-100 and BSA for 1 h. After incubated with primary antibody at 4 °C overnight and following the matched secondary antibody, the cells were visualized with a fluorescence microscope (Olympus, Tokyo, Japan).

For the Immunocytochemistry, tissue slides were deparaffinized and rehydrated. The tissue sections were incubated with primary antibodies overnight at 4 °C. Then, the sections were incubated with matched secondary antibody for 30 min at room temperature, stained with DAB, and counterstained with hematoxylin.

### Gene set enrichment analysis

Gene set enrichment analysis (GSEA) was performed to explore the pathways associated with SENP1 or CLDN6 in breast cancer. Gene expression profiles of breast cancer samples were acquired from GEO database. Tests were performed using GSEA v4.0 with permutation number set at 1000, and the threshold for the nominal *p* value was set to 0.05.

### Animal experiments

28 female BALB/c nude mice (6 weeks old) were purchased from SPF Biotechnology (Beijing, China) and were randomized into four groups. All animal experiments were conducted in accordance with the institutional guidelines and were approved by the Experimental Animal Ethical Committee of Jilin University. Cells were injected into the mice via the tail vein at a concentration of 1 × 10^6^ cells/0.1 mL PBS per mouse. After 4 weeks, the mice were injected with fluorescein sodium (150 mg/kg) and bioluminescence imaging was performed. Then the mice were euthanized, and the lungs were removed and fixed in 10% formalin. The lung metastatic nodules were examined macroscopically and subjected to hematoxylin and eosin (H&E) staining. Genomic DNA was extracted from peripheral blood in 5 mice per group and measured by qPCR assays with primers for human HK2 gene and mouse 18S rRNA to reflect the amount of circulating tumor cells [27].

### Tissue microarray and human clinical specimens

Tissue microarray was purchased from CN Alenabio http://www.alenabio.com (NO. BR1005b). The cohort contained 50 pairs of primary breast cancer tissues and matched lymphatic metastasis. Among them, 44 cases were invasive ductal carcinoma, 5 cases were invasive lobular carcinoma and 1 case was mucinous carcinoma. The staining was performed as described in Immunocytochemistry.

Fresh human normal breast tissues, breast cancer tissues and lymph node metastasis for Western blot analyses were collected from the second hospital of Jilin University. All samples were immediately frozen in liquid nitrogen after surgery and then later stored at − 80 °C for further use. The study was approved by the Ethics Committee of Jilin University and written consent was obtained from each patient.

### Proximity ligation assay

Proximity Ligation Assay (PLA) was performed to detect close proximity between CLDN6 and β-catenin. A similar double immunostaining protocol was performed, with the secondary antibodies replaced by secondary PLA probes from the Duolink kit (DUO92101, Sigma Aldrich, MA, USA). The assay was performed according to the manufacturer’s protocol. Hybridization between two PLA plus and minus probes leads to a fluorescent signal and occurs only when the distance between two antigens is no more than 40 nm.

### Statistical analysis

All statistical analyses were carried out using the SPSS 19.0 statistical software package (SPSS Inc., Chicago, IL, USA). The data were presented as the mean ± standard deviation (SD) at least three independent experiments. Data were analyzed using one-way analysis of variance or Student’s t-test for comparison between groups. The protein expression levels and clinicopathologic parameters were compared by χ2 test. The protein expression in primary breast cancer tissues and lymphatic metastasis was compared by paired t-test. Correlations between gene expression levels were calculated by Spearman’s rank correlation coefficients. *p* < 0.05 was statistically significant.

## Results

### HIF-1α is a potential upstream regulator of CLDN6

**Fig. 1:**
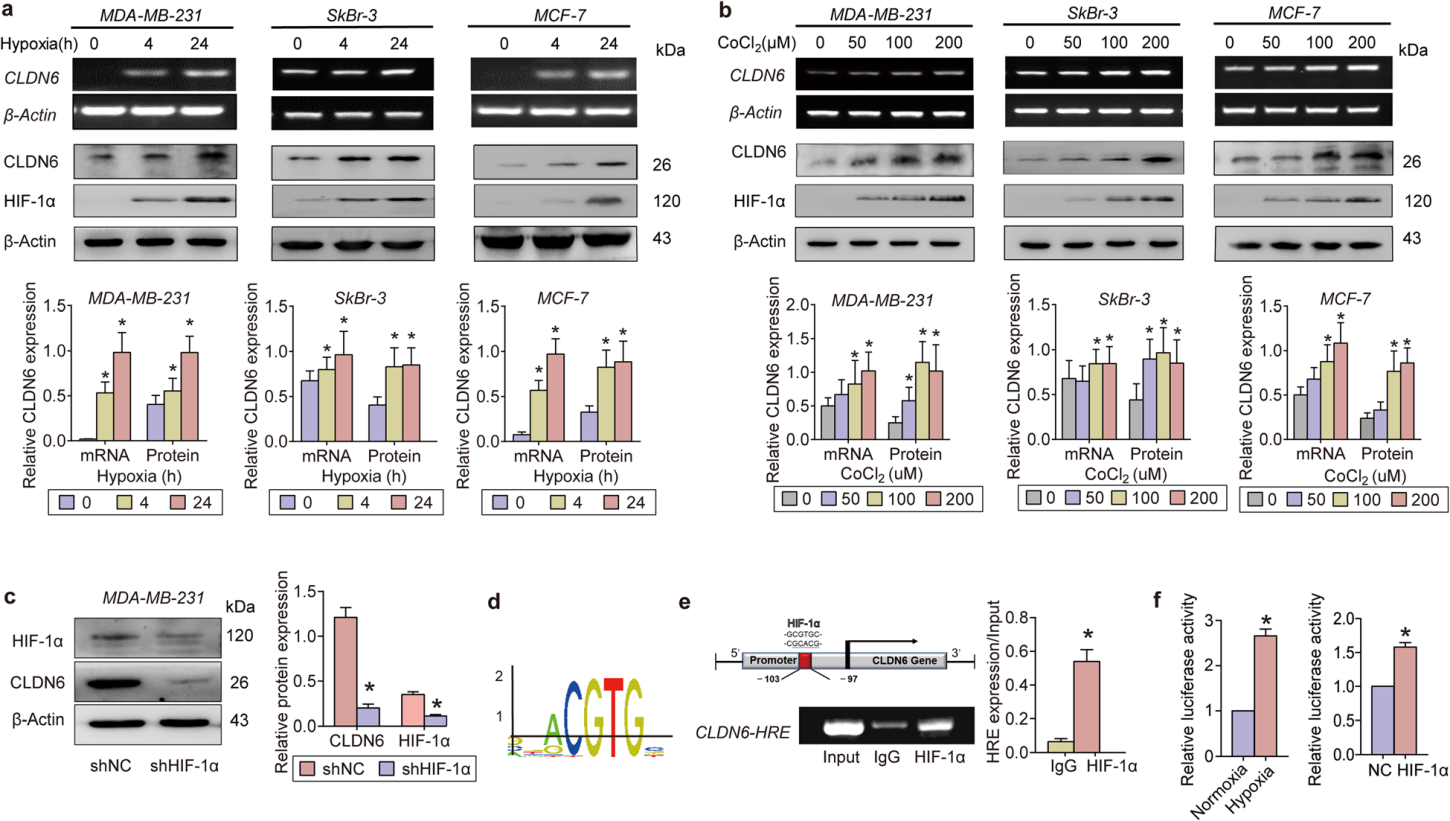
CLDN6 is directly upregulated by HIF-1α in breast cancer cells. **a** CLDN6 mRNA and protein levels under hypoxia in different molecular subtypes of breast cancer cells. **b** CLDN6 mRNA and protein levels after treatment with CoCl_2_ in different molecular subtypes of breast cancer cells. **c** WB analysis of HIF-1α and CLDN6 in MDA-MB-231 shNC or shHIF-1α cells under 1% O_2_ for 24 h. **d** Schematic diagram of the HRE sequences from the JASPAR database. **e** ChIP experiment to determine the combination of HIF-1α with the predicted HRE sequences in the CLDN6 promoter. * *p* < 0.05

Due to the differences in breast cancer cell lines with different molecular subtypes, three breast cancer cell lines were used to detect CLDN6 expression under hypoxia (MDA-MB-231: basal-like; MCF-7: luminal A; and SkBr-3: HER2+). Cells cultured under hypoxia for 4 or 24 h showed significantly increased CLDN6 expression at both the mRNA and protein levels (Fig. 1a). Because HIF-1α is widely known as the key transcription factor that mediates adaptive cellular hypoxia responses (Fig. 1a), we speculated that the hypoxia-induced increase in CLDN6 was mediated by HIF-1α. Cobalt dichloride (CoCl_2_) is a potent Fe^2+^ competitor that blocks the oxidative respiratory chain in cells and is thought to be an HIF-1α stabilizer based on its inhibition of PHD activity. Here, we treated cells with CoCl_2_ and observed that chemical hypoxia had the same effect on CLDN6 expression as low oxygen partial pressure conditions (Fig. 1b). HIF-1α RNAi lentiviruses were used to knock down hypoxia-induced HIF-1α accumulation, and they caused CLDN6 downregulation (Fig. 1c). These results suggested that HIF-1α upregulates CLDN6 expression under hypoxia.

HIF-1α regulates the hypoxia response by binding to HREs in target gene promoters (Fig. 1d), leading to the transcriptional activation of hundreds of genes. We searched for the promoter region of the human CLDN6 gene and identified an HRE with a strong binding possibility. ChIP assay was performed to investigate whether HIF-1α binds directly to the CLDN6 promoter and promotes CLDN6 transcription (Fig. 1e). Luciferase Report Assay showed that exposure to hypoxia or overexpression of HIF-1α significantly increased the fluorescence of PGl3-CLDN6 cells. These results suggest that HIF-1α is a potential upstream regulator of CLDN6.

### CLDN6 reduces breast cancer metastasis by inhibiting HIF-1α expression

**Fig. 2:**
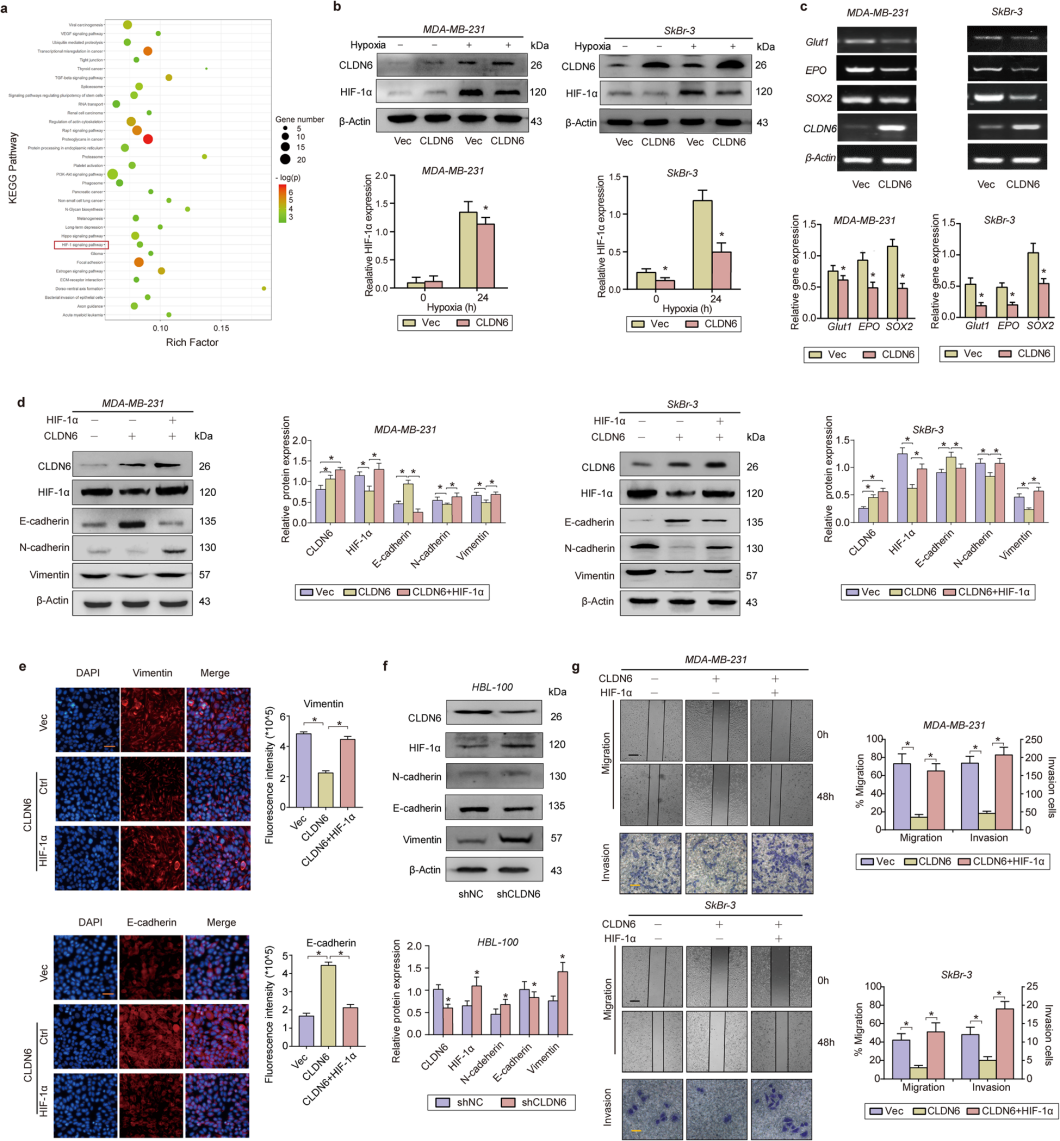
CLDN6 reduces breast cancer metastasis by inhibiting HIF-1α expression. **a** KEGG pathway analysis indicating that CLDN6 affects the HIF-1 signalling pathway. **b** WB analysis indicating that CLDN6 overexpression downregulates HIF-1α expression. **c** RT-PCR indicating that CLDN6 overexpression downregulates HIF-1α target gene expression. **d** WB analysis of EMT-related proteins in CLDN6-overexpressing and CLDN6/HIF-1α-overexpressing breast cancer cells under hypoxia for 24 h. **e** IF analysis of EMT-related proteins in CLDN6-overexpressing and CLDN6/ HIF-1α-overexpressing breast cancer cells. Scale: 100um. **f** WB analysis of HIF-1α and EMT-related proteins in CLDN6-KO HBL-100 cells. **g** Scratch and transwell invasion assays of CLDN6-overexpressing and CLDN6/HIF-1α-overexpressing breast cancer cells. Scale: 100um (above), 50um (below) * *p* < 0.05

We previously observed CLDN6 loss in breast cancer tissues, and functional experiments showed that CLDN6 inhibits EMT in cells [28]. Accordingly, CLDN6 has been described as the tumour suppressor gene in breast cancer. Using lentivirus transduction, we constructed a breast cancer MDA-MB-231 cell line with stable overexpression of CLDN6 (Additional file 1: Fig. S1). We next performed an mRNA microarray using MDA-MB-231/Vec and MDA-MB-231/CLDN6 cells to explore the possible role of CLDN6. Surprisingly, a KEGG analysis identified HIF-1 signalling as a pathway affected by CLDN6 overexpression, indicating that there may be a feedback mechanism involving CLDN6 and HIF-1α (Fig. 2a).

Considering the distinct function of CLDN6 and HIF-1α in tumour metastasis, we chose a breast cancer cell line with strong invasion, MDA-MB-231, and a breast cancer cell line with weaker invasion, SkBr-3, for the following experiments. We first detected HIF-1α expression in CLDN6-overexpressing breast cancer cells and found that CLDN6 significantly decreased HIF-1α accumulation under hypoxia (Fig. 2b). Next, we randomly measured the mRNA levels of three HIF-1α target genes and found that CLDN6 decreased *Glut1, EPO* and *SOX2* expression under hypoxia (Fig. 2c). Moreover, the Western blot assay showed that CLDN6 inhibit the expression of Glut1, EPO and SOX2 in protein level as well (Fig. s2), indicating that CLDN6 impairs the transcriptional activity of HIF-1α.

The TCGA database showed that CLDN6 expression was positively correlated with E-cadherin (Fig. s3a) and negatively correlated with vimentin (Fig. s3b). In addition, E-cadherin was upregulated in CLDN6-overexpressing cells following the decreases in N-cadherin and vimentin, indicating that CLDN6 inhibits EMT. However, the restoration of HIF-1α rescued the inhibition of EMT induced by CLDN6 overexpression (Fig. 2d, Fig. 2e). CLDN6 knockdown in the normal human epithelial cell line HBL-100 increased HIF-1α expression and promoted EMT (Fig. 2f). Besides, the restoration of HIF-1α rescued the inhibition of migration and invasion induced by CLDN6 overexpression (Fig. 2g). These results suggest that CLDN6 reduced EMT and tumour metastasis by inhibiting HIF-1α under hypoxia.

### CLDN6 promotes HIF-1α proteolysis via the VHL/PHD-independent pathway

**Fig. 3:**
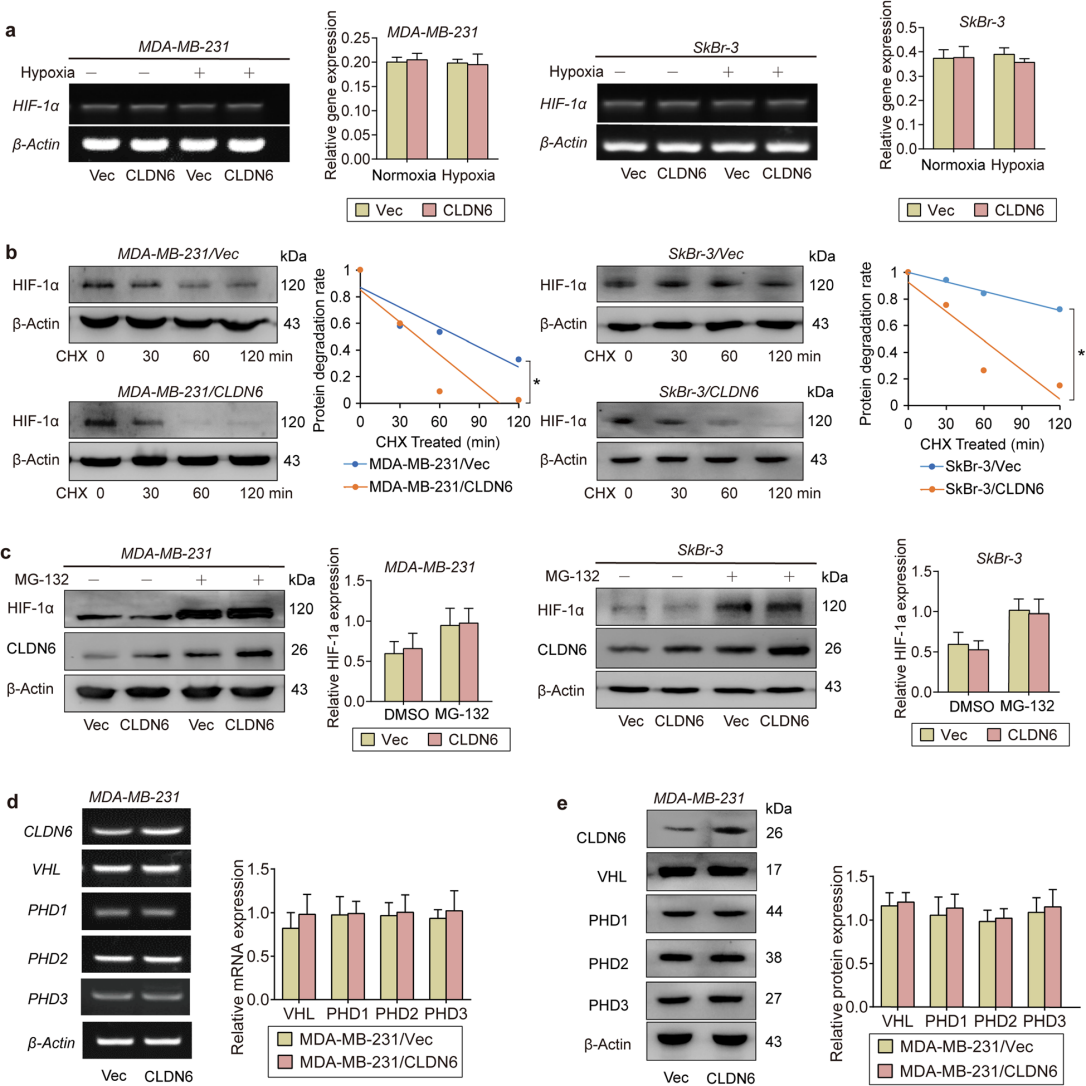
CLDN6 promotes HIF-1α degradation via the VHL/PHD-independent pathway. **a** RT-PCR indicated that CLDN6 overexpression had no significant effect on HIF-1α mRNA expression. **b** Evaluation of the HIF-1α degradation rate after treating hypoxia cells with CHX for 0, 30, 60 or 120 min. **c** Cells were treated with MG-132 to assess whether HIF-1α is degraded by the ubiquitin-proteasome pathway in normoxia conditions. **d** mRNA levels of VHL, PHD1, PHD2 and PHD3 in MDA-MB-231/Vec and MDA-MB-231/CLDN6 cells. **e** Protein levels of VHL, PHD1, PHD2 and PHD3 in MDA-MB-231/Vec and MDA-MB-231/CLDN6 cells. * *p* < 0.05

Since CLDN6 inhibited HIF-1α protein accumulation under hypoxia, we next detected whether HIF-1α was also altered at the transcriptional level. RT-PCR showed that CLDN6 did not affect HIF-1α mRNA expression in either MDA-MB-231 or SkBr-3 cells (Fig 3a), indicating that CLDN6 regulates HIF-1α at the post-transcriptional level. We hypothesized that CLDN6 affects the protein stability of HIF-1α and detected the half-life of the HIF-1α protein by treating cells with cycloheximide (CHX), a protein synthesis inhibitor. CLDN6 shortened the half-life of the HIF-1α protein (Fig. 3b) and MG-132, a proteasome inhibitor, eliminated the inhibition of HIF-1α, indicating that CLDN6 promotes HIF-1α degradation via the ubiquitin-proteasome pathway (Fig. 3c).

It is widely known that HIF-1α expression is tightly regulated by the classic VHL-PHD pathway. PHDs (including PHD1–3) hydroxylate the amino acids 402 and 564 of HIF-1α and trigger a ubiquitination reaction with VHL, leading to HIF-1α degradation. However, we did not detect any significant changes in VHL or PHDs at either the mRNA or protein level (Fig. 3d, e), indicating that CLDN6 promotes HIF-1α proteolysis via a VHL/PHD-independent pathway.

### CLDN6 inhibits HIF-1α deSUMOylation by downregulating SENP1

SUMOylation of HIF-1α serves another signal for VHL binding for ubiquitin-dependent degradation, even under hypoxia. Accumulating evidence has revealed that as an essential catalyst that deSUMOylates SUMO-conjugated proteins, SENP1 is involved in activation of the hypoxic response and stabilization of HIF-1α.

**Fig. 4:**
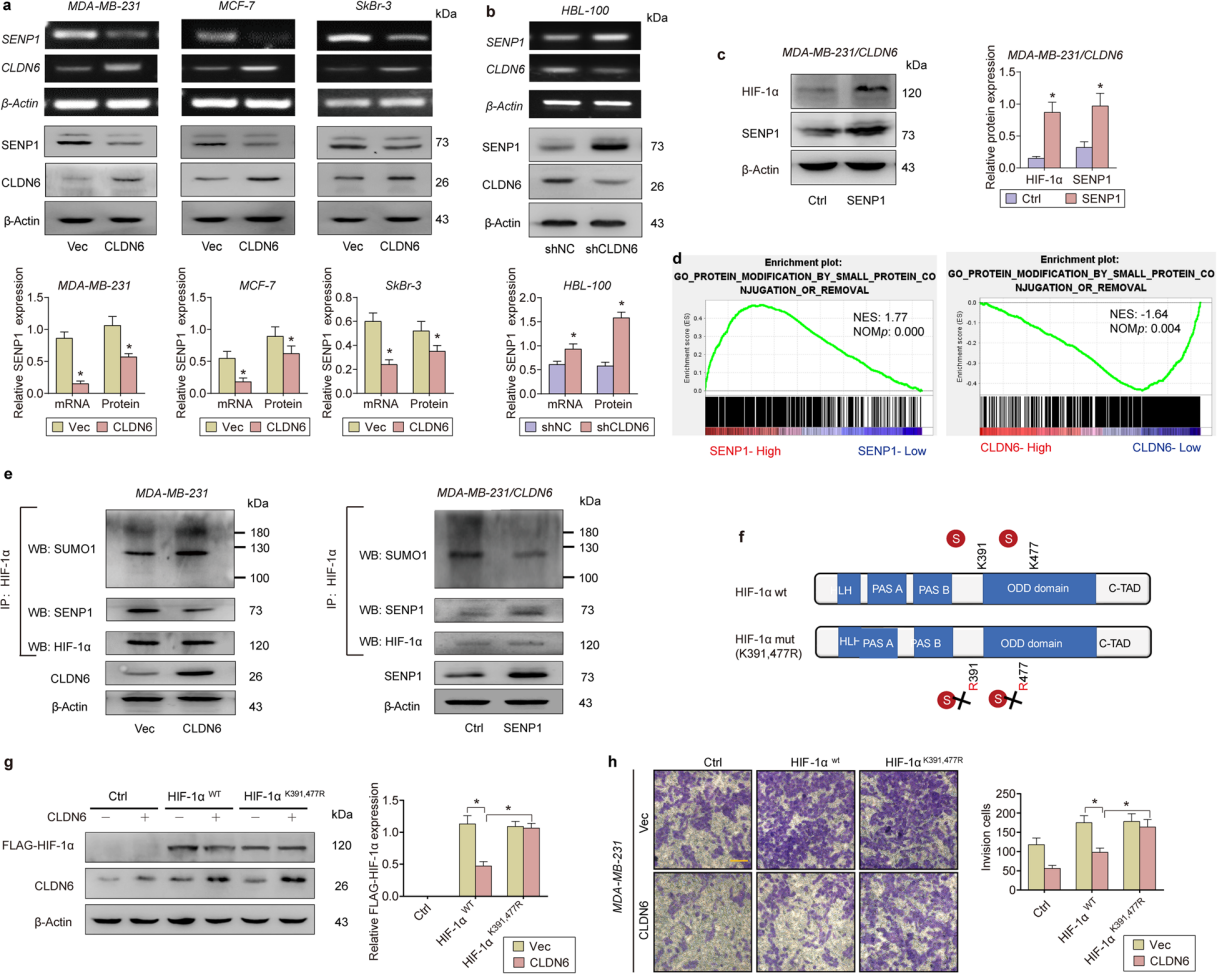
CLDN6 inhibits HIF-1α deSUMOylation by downregulating SENP1. **a** SENP1 mRNA and protein levels in CLDN6-overexpressing breast cancer cells. **b** SENP1 mRNA and protein levels in CLDN6-KO HBL-100 cells. **c** SENP1 overexpression in MDA-MB-231/CLDN6 cells restored the expression of HIF-1α. **d** GSEA plots of the Protein Modification by Small Protein Conjunction Or Removal pathway analysed according to SENP1 (left) or CLDN6 (right) expression. **e** Comparison of SUMO1-HIF-1α binding levels between CLDN6-overexpressing and CLDN6/SENP1-overexpressing MDA-MB-231 cells. Cells were treated with proteasome inhibitor MG-132, so that there was the same amount of background HIF-1α expression. **f** Map of the SUMOylation site mutant HIF-1α plasmid. **g** WB analysis indicating that CLDN6 downregulated WT but not MUT HIF-1α expression. **h** Mutation of the SUMOylation sites in HIF-1α rescued the loss of metastasis in CLDN6-overexpressing breast cancer cells. Scale: 50um. * *p* < 0.05

The mRNA sequencing results showed that CLDN6 significantly reduced the expression of *SENP1* but not that of five other *SENP* family members (Fig. s4a). A decrease in SENP1 was also found in CLDN6-overexpressing breast cancer cell lines at both the mRNA and protein levels (Fig. 4a). Consistently, SENP1 was upregulated in CLDN6 KO HBL-100 cells (Fig. 4b). The overexpression of SENP1 in MDA-MB-231/CLDN6 cells restored HIF-1α expression, indicating that CLDN6 inhibits HIF-1α accumulation by downregulating SENP1 (Fig. 4c).

We next examined whether the CLDN6/SENP1 axis affects the SUMOylation of HIF-1α. Gene expression data from a breast cancer microarray were acquired from the GEO database (GSE27562), and a GSEA was performed for SENP1 and CLDN6 expression. The enrichment plots indicated that SENP1 expression exhibited significant positive relationships with the *Protein Modification by Small Protein Conjunction or Removal pathway* genes, whereas CLDN6 expression had a negative relationship with these genes (Fig. 4d). In addition, SENP1 overexpression restored the increased global SUMOylation in MDA-MB-231/CLDN6 cells (Fig. s4b, Fig. s4c). An IP experiment showed that CLDN6 overexpression increased the SUMO-1 conjunction of HIF-1α and SENP1 restored the upregulated SUMOylation in MDA-MB-231/CLDN6 cells (Fig. 4e), indicating that CLDN6 inhibited HIF-1α deSUMOylation by reducing SENP1 expression.

Because K391 and K477 SUMO sites are required for SENP1-regulated HIF-1α deSUMOylation, we wondered whether mutations in the SUMO site in HIF-1α would prevent the effect of CLDN6 overexpression in breast cancer cells. To test this, we generated a HIF-1α ^K391R/K477R^ plasmid that lacks the ability to bind SUMO1 (Fig. 4f). A co-transfection experiment confirmed that SENP1 overexpression increased the level of HIF-1α ^WT^ but not HIF-1α ^K391/K477R^ (Fig. s4d). More impressively, CLDN6 inhibited the accumulation of HIF-1α ^WT^ but not HIF-1α ^K391/K477R^ (Fig. 4g). A transwell invasion assay was performed and showed that mutating the SUMO sites in HIF-1α rescued the loss of metastasis in MDA-MB-231/CLDN6 cells (Fig. 4h). Notably, both HIF-1α ^WT^ and HIF-1α ^K391/K477R^ could significantly increase the expression of CLDN6, which is consistent with our previous results.

### CLDN6 inhibits breast cancer metastasis through SENP1/HIF-1 signalling in vivo

**Fig. 5:**
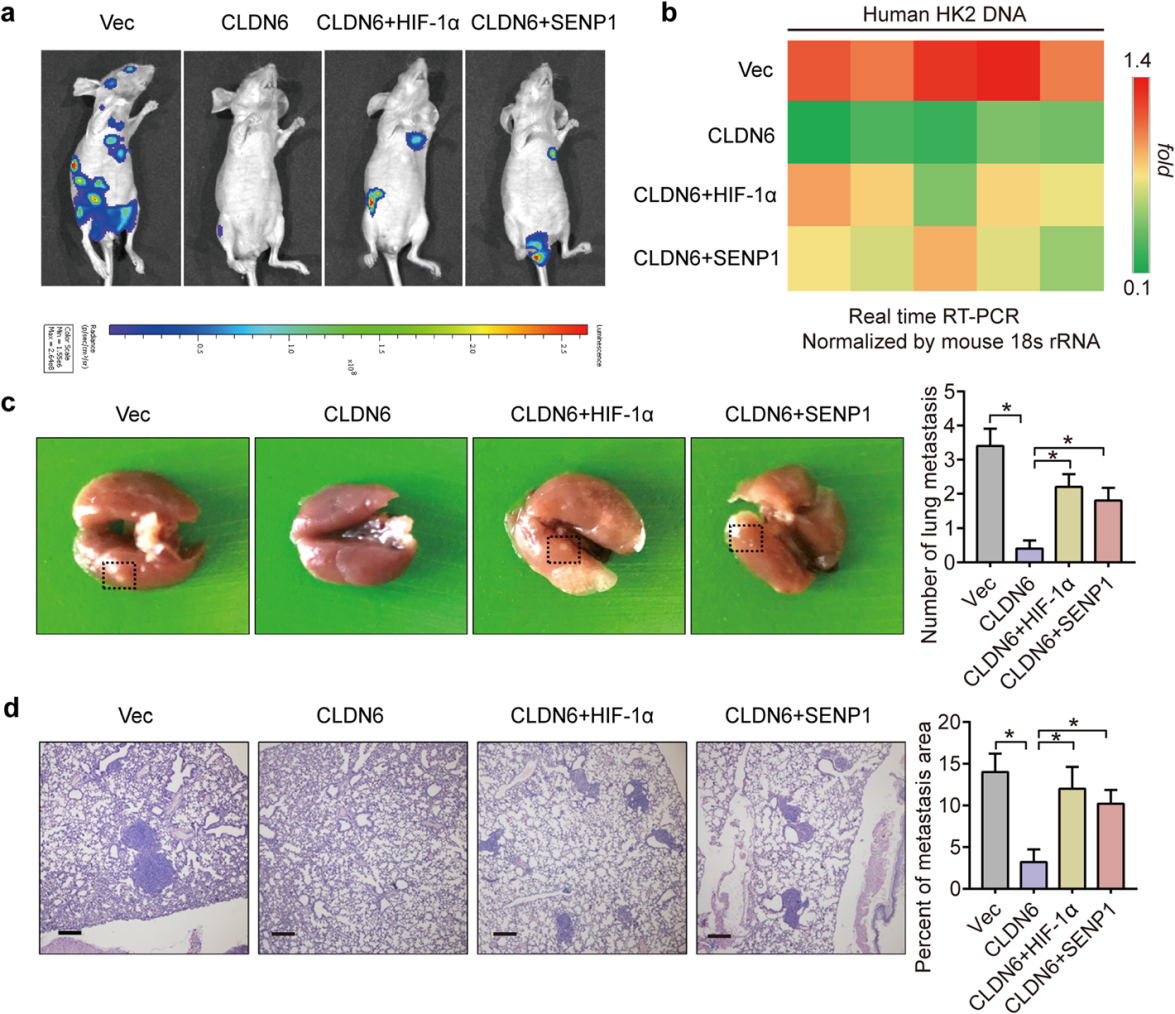
CLDN6 inhibits breast cancer metastasis through SENP1/HIF-1α signalling in vivo. **a** Bioluminescence analysis of lung metastasis derived from MDA-MB-231 cells. **b** The ratio of the human HK2 gene to mouse 18S rRNA in genomic DNA from mouse peripheral blood was used to reflect the amount of circulating tumour cells. **c** Representative examples of lungs with metastatic foci are shown, and the average number of lung metastases in each group is shown. **d** Representative HE staining of lung sections is shown. Scale: 100um. * *p* < 0.05

To further determine the functional roles of the CLDN6/SENP1/HIF-1α axis in breast cancer metastasis, MDA-MB-231 cells carrying the luciferase gene were stably transfected with CLDN6 overexpression, CLDN6/HIF-1α overexpression, CLDN6/SENP1 overexpression and corresponding empty vectors and injected into nude mice. The luciferase images (Fig. 5a), lung metastatic nodules (Fig. 5c) and HE staining of lung tissues (Fig. 5d) showed that overexpression of SENP1 or HIF-1α significantly restored the CLDN6-mediated reductions in lung metastasis. In addition, we collected peripheral blood from the mice and extracted the genomic DNA. The human HK2 gene was evaluated by real-time PCR and normalized to mouse 18S to reflect the amount of circulating tumour cells in the peripheral blood of mice (Fig. 5b). Consistently, the overexpression of SENP1 or HIF-1α significantly restored the CLDN6-mediated reductions in circulating tumour cells, indicating that CLDN6 inhibits breast cancer metastasis through SENP1/HIF-1α signalling in vivo.

### CLDN6 is lost in lymphatic metastasis and inversely associated with HIF-1α expression

**Fig. 6:**
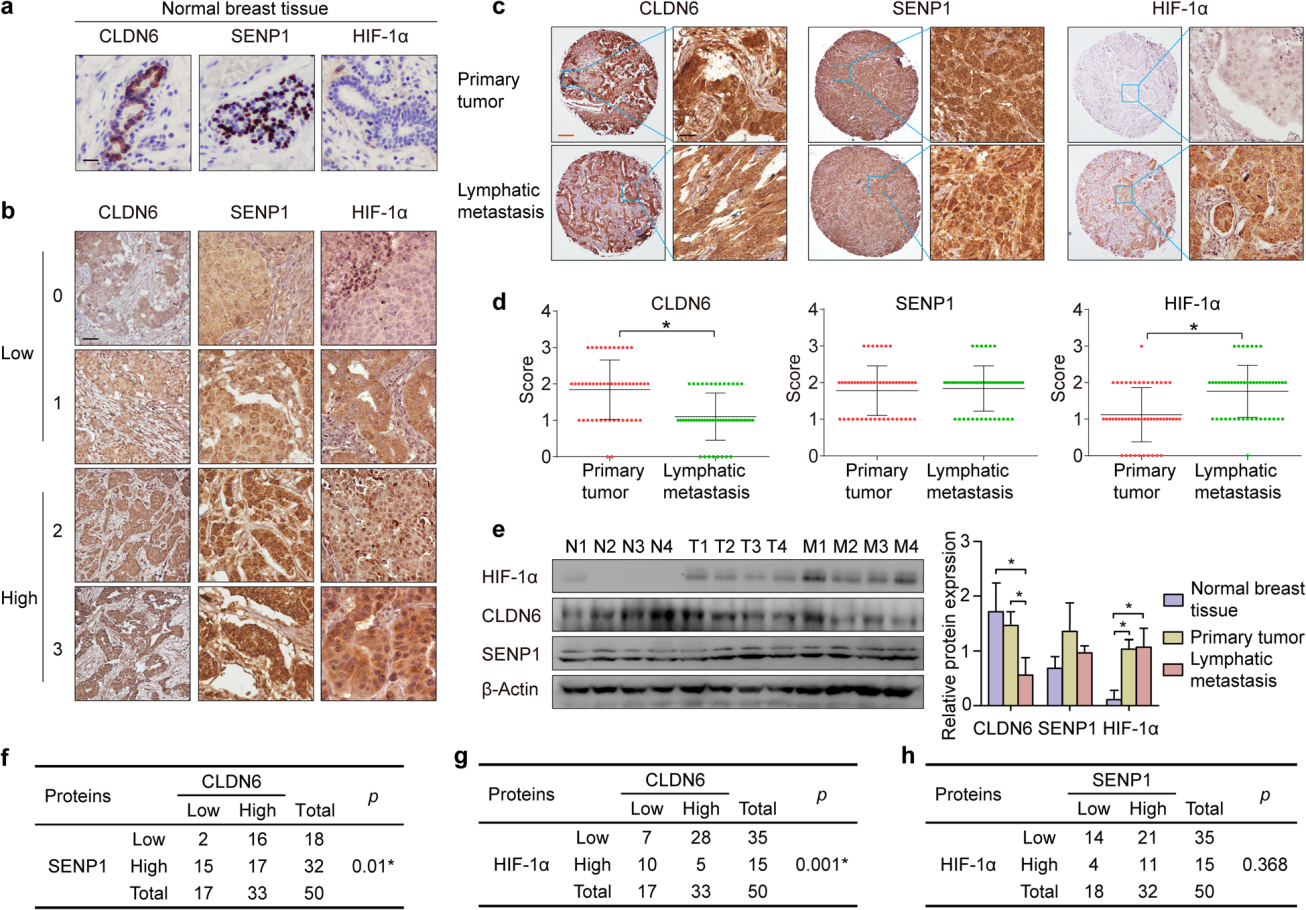
CLDN6 is lost in lymphatic metastasis and negatively associated with SENP1 and HIF-1α expression. **a** Immunohistochemical staining for CLDN6, SENP1 and HIF-1α expression in normal breast tissues. Scale: 20um. **b** Immunohistochemical staining for CLDN6, SENP1 and HIF-1α expression in breast cancer tissues. Scale: 20um. **c** Representative images showing CLDN6, SENP1 and HIF-1α staining in primary breast cancer tissues and matched lymphatic metastasis tissues. Scale: 200um (left), 20um (right). **d** CLDN6, SENP1 and HIF-1α IHC staining scores in primary breast cancer tissues (*n* = 50) and lymphatic metastasis tissues (n = 50) were shown. **e** Western blot of CLDN6, SENP1 and HIF-1α expression in random selected each 4 cases of normal breast tissues (N), breast cancer tissues (T) and lymph node metastasis (M). **f** Correlation between CLDN6 and HIF-1α protein expression in breast cancer tissues. **g** Correlation between CLDN6 and SENP1 protein expression in breast cancer tissues. **h** Correlation between SENP1 and HIF-1α protein expression in breast cancer tissues. * *p* < 0.05

To validate the relationship between CLDN6/SENP1/HIF-1α signalling and metastasis in breast cancer patients, a paraffin-embedded tissue array containing 50 paired primary/lymphatic metastatic clinical breast cancer specimens was obtained. In addition, 10 cases of normal breast tissues from the second hospital of Jilin University was collected and stained as the location control (Fig. 6a). CLDN6 was mainly expressed in the cell membrane of normal breast cells, while SENP1 was located in the nucleus. Consistent with previous reports, HIF-1α is difficult to detect in normal breast tissues. IHC staining of the tissue array was scored as 0, 1, 2, or 3 according to the intensity (Fig. 6b). A staining score of 0 or 1 was considered to be low expression, while a score of 2 or 3 was considered to be high expression. Using a paired T-test, we found that the HIF-1α score was significantly higher in lymphatic metastasis tissues than in primary lesions (Fig. 6c, d), which is consistent with the conclusion of previous studies that HIF-1α is closely related to tumour metastasis. Notably, the CLDN6 score was significantly lower in lymphatic metastasis tissues than in primary breast cancer tissues, and a consistent conclusion was reached in the Western blotting of CLDN6 in different tissues (Fig. 6e), indicating that CLDN6 loss was positively associated with lymphatic metastasis in breast cancer. The Western blotting also showed that the expression of HIF-1α in lymph node metastasis was higher than that in in-situ cancer tissues, but the difference was not statistically significant (Fig. 6e), which may be related to the small number of samples.

A Pearson correlation analysis demonstrated that CLDN6 expression was negatively correlated with SENP1 (Fig. 6f) and HIF-1α (Fig. 6g) in breast cancer tissues. The detailed results of the clinicopathological correlation analysis are described in the SUPPLEMENTAL MATERIAL: Tables s1, s2 and s3. Thus, we propose that CLDN6 plays an anti-metastatic role in breast cancer by antagonizing the SENP1/HIF-1α signalling pathway.

### CLDN6 downregulates SENP1 expression by blocking the nuclear translocation of β-catenin

**Fig. 7:**
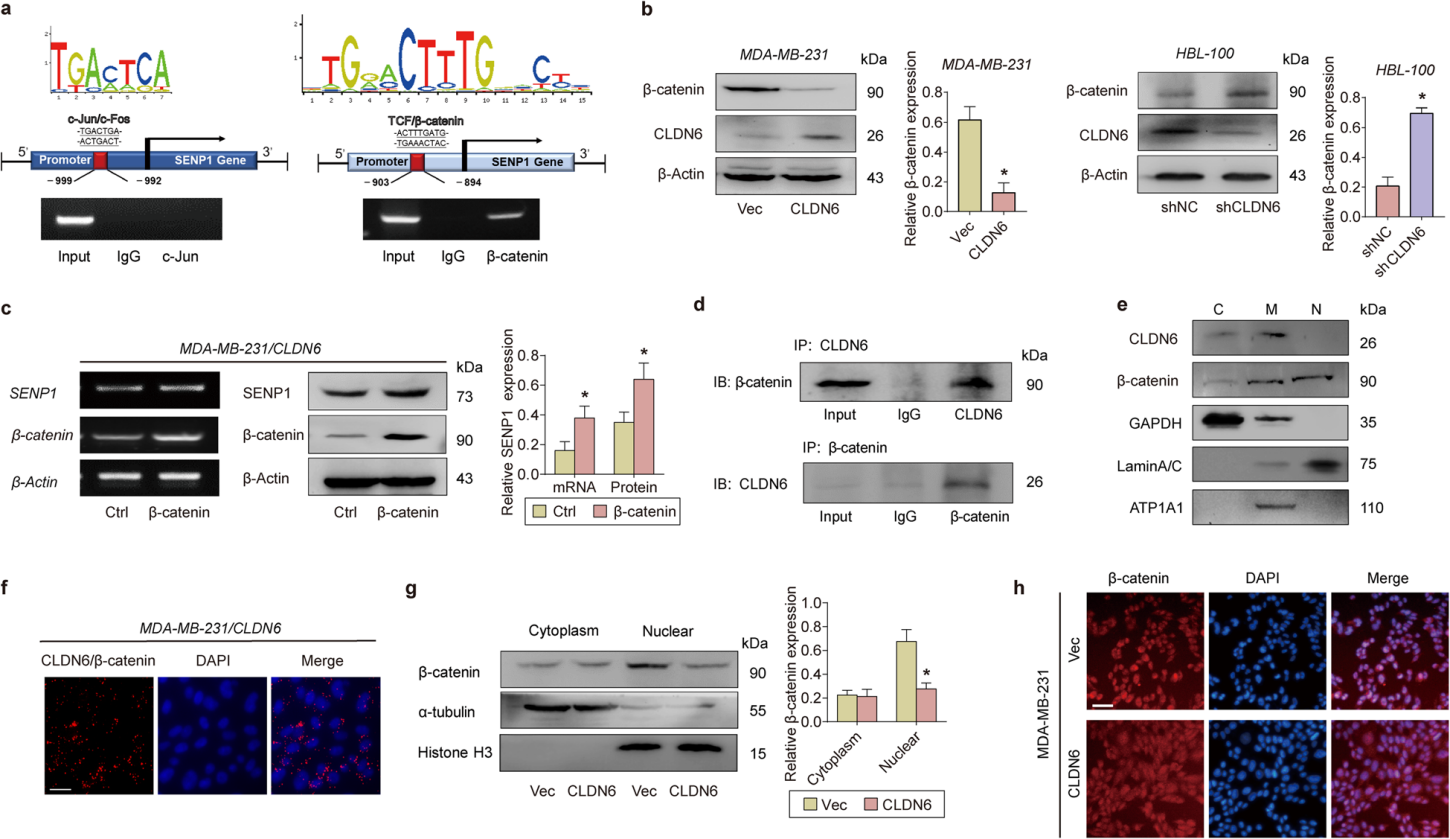
CLDN6 downregulates SENP1 expression by blocking the nuclear translocation of β-catenin. **a** ChIP experiment of the combination of c-Jun and β-catenin to the predicted sequences in the SENP1 promoter. **b** β-catenin mRNA and protein levels in CLDN6-overexpressing MDA-MB-231 cells and CLDN6-KO HBL-100 cells. **c** β-catenin overexpression rescued SENP1 expression in MDA-MB-231/CLDN6 cells. **d** Co-IP experiment indicated that CLDN6 and β-catenin combined with each other in MDA-MB-231/CLDN6 cells. **e** Sequential fractionation to determine the location of CLDN6 and β-catenin. The cytosolic, membrane bound organelles and nuclear fractions are denoted by C, M and N, respectively. **f** PLA showed the co-localization of CLDN6 and β-catenin in MDA-MB-231/CLDN6 cells. Scale:100um. **g** WB analysis of β-catenin extracted from the cytoplasm and nucleus. **h** IF indicating that CLDN6 promotes β-catenin cytoplasm translocation. Scale:50um. * *p* < 0.05

Currently, the regulatory mechanism of SENP1 is unclear. Because CLDN6 downregulated SENP1 expression at the transcriptional level, we speculated that this process may be mediated by the transcription factors of SENP1. We used the transcription factor prediction site GCBI (https://www.gcbi.com.cn/) to predict two promising binding sites in the promoter of SENP1: the AP1(c-Jun/c-Fos dimer) binding site and the TCF/β-catenin binding site. ChIP assays showed that β-catenin binds to the predicted site but not c-Jun, indicating that β-catenin may be a transcription factor of SENP1 (Fig. 7a). The TCGA breast cancer database showed that there was a significant positive correlation between the expression of β-catenin and SENP1 (Fig. s5a). We also observed that β-catenin was downregulated in MDA-MB-231/CLDN6 cells (Fig. 7b), and its restoration eliminated the inhibition of SENP1 induced by CLDN6 overexpression (Fig. 7c).

Like CLDN6, β-catenin is also a member of the TJ structure. Because β-catenin can interact with a variety of PDZ domains [29] and these interactions affect the localization and activity of β-catenin, we examined whether CLDN6 interacts with β-catenin and has an effect on its biological role. Co-IP (Fig. 7d) experiment revealed that CLDN6 and β-catenin bind to each other in MDA-MB-231/CLDN6 cells. Subcellular fractionation showed that CLDN6 expressed in the cell membrane and cytoplasm, while β-catenin mainly expressed in the nucleus, but also in the cytoplasm and cell membrane (Fig. 7e), suggesting that CLDN6 may bind to β-catenin in cytoplasm and membrane. Further, we used proximity ligation assay (PLA) to detect whether there is a direct intermolecular interaction between CLDN6 and β-catenin. MDA-MB-231/CLDN6 cells were fixed and incubated with anti-CLDN6 (Rabbit) and anti-β-catenin (Mouse) antibodies, followed by Duo link reaction. The results showed that CLDN6 and β-catenin directly interacted in the cytoplasm of cancer cells (Fig. 7f). CLDN6 reduced β-catenin expression in the nucleus rather than in the cytoplasm (Fig. 7g). We also observed the cytoplasmic translocation of β-catenin in CLDN6-overexpressing cells by IF (Fig. 7h). The results indicated the possibility that CLDN6 binds to β-catenin in the cytosol and blocks its nuclear translocation. Protein half-life experiments showed that CLDN6 promoted β-catenin degradation (Fig. s5b) by the ubiquitin pathway (Fig. s5c). These results indicated that in the cytoplasm, CLDN6 combines and retains β-catenin, causing the degradation of β-catenin and its inability to translocate to the nucleus.

## Discussion

Tumour hypoxia triggers a set of adaptive responses that ultimately promote a more aggressive tumour phenotype and are primarily controlled by the transcription factor system of HIFs. HIF-1α functions as a master regulator of hypoxia-mediated tumour metastasis mainly by promoting EMT through directly upregulating TWIST and SNAI expression [30]. As a result of these diverse functions of its downstream genes, HIF-1α has been widely recognized as a target for cancer therapy [31, 32].

Regulation of the homeostasis system requires the participation of internal feedback mechanisms, such as the widely known p53/mdm2 feedback loop. Mdm2 is transcriptionally induced by p53 but in return blocks p53 activity, forming a negative feedback circuit to inhibit the profoundly adverse effect of p53 on cell growth and proliferation [33]. However, these molecules do not act alone, as there is increasing evidence that similar feedback mechanisms exist among HIF-1α and its targets [34]. Here, we have discovered a new downstream gene of HIF-1α, CLDN6, which also participates in oxygen homeostasis regulation through negative feedback mechanism by regulating HIF-1α stability.

CLDN6 is an integral component of TJ proteins and plays a crucial role in maintaining cell integrity. CLDN6 loss in tumour tissues correlates with tumour metastasis and poor prognosis. It is counterintuitive that CLDN6 is induced by HIF-1α under hypoxic conditions. However, a KEGG analysis identified HIF-1 signalling as a pathway affected by CLDN6, indicating that there may be a feedback mechanism involving CLDN6 and HIF-1α. This hypothesis was verified by the results showing that CLDN6 inhibited the accumulation and transcriptional activity of HIF-1α under hypoxia. The CLDN6-overexpressing breast cancer cell lines had significantly weaker migratory and invasive abilities both in vitro and in vivo. However, the inhibited metastasis was rescued by restoring HIF-1α in the CLDN6-overexpressing breast cancer cell lines. Our data support the hypothesis that breast cancer patients with primary CLDN6 loss are more likely to have tumour metastasis due to lack of feedback mechanism to inhibit HIF-1α stability. Immunohistochemical staining of clinical samples showed that CLDN6 was lowly expressed in lymphatic metastasis tissues, suggesting that CLDN6 loss may promote tumour metastasis. However, the ability of CLDN6 as a prognostic marker for breast cancer remains to be discussed, as our findings suggest that there seems no clinicopathological correlation of CLDN6 expression in human breast cancers. It should be noted that another key effector factor that mediates hypoxia adaption, HIF-2α, also contributes to breast cancer metastasis. Unlike HIF-1α mediated short-term acute hypoxia, HIF-2α is generally thought to play a role in long-term hypoxia [35]. And there is currently an evidence that HIF-2α is a substrate for SUMOylation in Hela cells [36]. Although CLDN6 significantly reduced the expression of SENP1, the accumulation of HIF-2α under hypoxia was not decreased in MDA-MB-231/CLDN6 cells (Fig. s6). Our data strongly supports the role of CLDN6 in mediating hypoxia-induced tumour metastasis, at least partially by mediating HIF-1α. However, the role of HIF-2α should still be taken into account in subsequent studies of chronic hypoxia.

SUMOylation, meaning the conjugation of SUMO to target proteins, has attracted increasing attention as a widely used post-translational protein modification. There are three types of SUMO isoforms in mammals, SUMO1–3 [37]. SUMO targets are located mainly in the nucleus, and SUMOylation of these substrates can alter their cellular localization, protein stability and biological activity [38, 39]. Although we have verified that CLDN6 inhibits SENP1 expression, our data are not sufficient to prove that CLDN6 inhibits HIF-1α by affecting its SUMOylation. However, the HIF-1α SUMO site mutant showed the ability to escape degradation by CLDN6, suggesting that SUMOylation plays a strong role in CLDN6-induced HIF-1α inhibition. The deSUMOylation enzyme SENP1 has recently been shown to have a prooncogenic role in cancer; however, our mechanistic understanding of how SENP1 is regulated is limited. In this study, β-catenin was identified as a transcription factor of SENP1. Although c-Jun was also predicted to bind to the promoter of SENP1, it was not verified in our ChIP assay. However, for β-catenin, we obtained satisfactory results. β-catenin was repressed by CLDN6, even under normal oxygen conditions, which may explain why CLDN6 inhibits the invasion and migration of breast cancer cells under both normoxic and hypoxic conditions.

β-Catenin is also a well-known component of adherens junctions. β-Catenin located at the plasma membrane interacts with E-cadherin to regulate cell adhesion, but in the cytoplasm, it participates in Wnt signalling; when located in the nucleus, it acts as a transcriptional activator [40]. β-catenin interacts not only with E-cadherin but also with some PDZ domain-containing proteins [41]. There has been increasing evidence suggesting that CLDNs can affect β-catenin. The loss of CLDN3 induces Wnt/β-catenin activation in colon cancer [42], while downregulation of CLDN4 results in E-cadherin loss and increased β-catenin signalling [43]. Our study showed the role of SENP1 in the inhibition of HIF-1α by CLDN6, but the contribution of other factors such as β-catenin should not be ignored. Studies have shown that β-catenin binds HIF-1α directly and enhances HIF-1-mediated transcription [44, 45]. Therefore, our data only provide a possible mechanism to explain the role of CLDN6 in hypoxia-induced breast cancer metastasis, and it is still necessary to explore further.

**Fig. 8:**
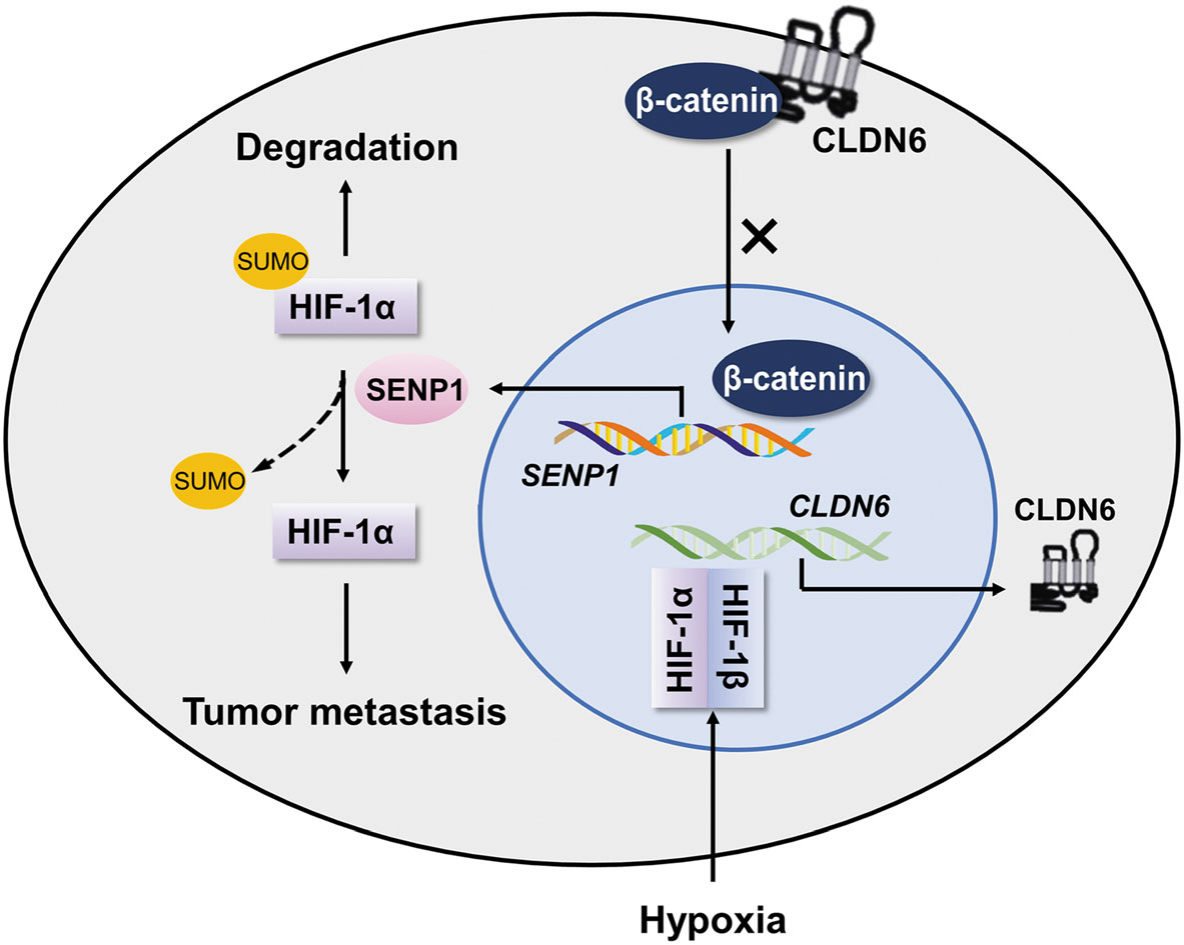
Proposed model of the CLDN6/HIF-1α negative regulatory feedback mechanism. HIF-1α is stabilized under hypoxia and heterodimerises with HIF-1β to bind to the HRE sequences within the promoter of CLDN6, which is increased at both the mRNA and protein levels. In the cytoplasm, CLDN6 combines and retains β-catenin, a transcription factor of SENP1, causing the degradation of β-catenin and its inability to translocate to the nucleus, which reduces SENP1 expression and prevents the deSUMOylation of HIF-1α, ultimately leading to HIF-1α degradation and breast cancer metastasis suppression

The loss of claudins seems to be associated with poor survival in breast cancer [46], and claudin-low has been classified as a molecular subtype of breast cancer that exhibits low expression of luminal markers and high levels of mesenchymal markers [47]. However, current studies indicate that some members of the claudin family, such as CLDN4 [48] and CLDN1 [49], play an oncogenic role in some types of tumours. Furthermore, as a member of the claudin family, the function of CLDN6 varies among different types of tumours [50, 51]. In hepatocellular carcinoma, CLDN6 is highly expressed and associated with poor prognosis (unpublished research by our group). This evidence indicates that disorders involving claudin family members have a possible impact on tumourigenesis. In our study, CLDN6 appears to be able to regulate the HIF-1 pathway through SENP1 not only in breast cancer cell lines but also in non-tumorigenic HBL-100 cell lines. Compared with breast cancer cell lines, HBL-100 cells express more CLDN6, and knocking down the expression of CLDN6 can promote interstitial-like transformation of HBL-100 cells. This may mean that CLDN6 is involved in multiple steps of the malignant process of mammary epithelial cells-maintaining the barrier function of normal mammary epithelial cells, inhibiting mesenchymal-like transformation of mammary epithelial cells, enhancing cell-to-cell adhesion, and weakening cell mobility. CLDN6 plays an important role in the migration, invasion and metastasis of breast cancer cells, but the exact underlying mechanism remains unclear. As a summary of our findings (Fig. 8), the HIF-1 dimer translocates to the nucleus and promotes the transcription of CLDN6 under hypoxia. CLDN6 retains β-catenin in the cytoplasm and reduces its nuclear translocation, thereby decreasing the expression of its downstream gene SENP1. The reduction in SENP1 promotes the SUMOylation of HIF-1α, which decreases the stability and transcriptional activity of HIF-1α, thus contributing to the inhibition of breast cancer metastasis. Our findings are significant for the aetiology of CLDN6 loss in breast cancers. CLDN6 has a potential role in the diagnosis and prognosis of cancers involving deregulated HIF-1α, and subsequently HIF-1α/SENP1-blocked therapies.

## Conclusion

In summary, this study provides new evidence for the clinical and biological significance of CLDN6 in breast cancer. In this study, we demonstrated a previously unrecognized negative feedback loop in which CLDN6 is transcriptionally upregulated by HIF-1α and in return reduces HIF-1α stability by inhibiting its deSUMOylation process via SENP1. Because CLDN6 attenuates hypoxia-induced tumour metastasis in a SUMOylation-dependent manner, these findings might provide a novel strategy for breast cancer treatment, and directly targeting SENP1/HIF-1α might prove to be a beneficial anti-cancer therapy.

## Supplementary information

**Additional file 1 Fig. S1.** Construction of MDA-MB-231 cell line with stable overexpression of CLDN6. **Fig. S2.** Western blotting indicating that CLDN6 overexpression downregulates HIF-1α target gene expression. **Fig. S3.** The correlation between CLDN6 and EMT factors. **Fig. S4.** CLDN6 inhibits HIF-1α deSUMOylation by down-regulating SENP1. **Fig. S5**. CLDN6 down-regulates SENP1 expression by blocking the nuclear translocation of β-catenin. **Fig. S6**. CLDN6 does not affect the expression of HIF-2α. **Table S1.** Clinicopathological correlation of CLDN6 expression in human breast cancers. **Table S2.** Clinicopathological correlation of SENP1 expression in human breast cancers. **Table S3.** Clinicopathological correlation of HIF-1α expression in human breast cancers.

## Data Availability

The datasets used and/or analyzed during the current study are available from the corresponding author on reasonable request.

## Abbreviations

ChIP: Chromatin immunoprecipitation assay
CHX: Cycloheximide
CLDN6: Claudin6
DMEM: Dulbecco’s modified Eagle’s medium
EMT: Epithelial-mesenchymal transition
GSEA: Gene set enrichment analysis
HE: Hematoxylin and eosin
HIF-1: Hypoxia-inducible factor 1
HREs: Hypoxia response elements
IF: Immunofluorescence
IHC: Immunohistochemistry
KEGG: Kyoto encyclopedia of genes and genomes
KO: Knockdown
NC: Negative control
PHDs: Prolyl hydroxylase domain enzymes
PLA: Proximity ligation assay
SD: Standard deviation
SENP1: SUMO/sentrin-specific peptidase 1
SUMO: Small ubiquitin-related modifier protein
TCGA: The cancer genome atlas
TJ: Tight junctions
VHL: Von Hippel-Lindau
