# Supplementary material for: A SUMOylation-dependent HIF-1α/CLDN6 negative feedback mitigates hypoxia-induced breast cancer metastasis
Source: J Exp Clin Cancer Res. 2020 Feb 24;39:42. doi: 10.1186/s13046-020-01547-5 (PMC7038627; doi:10.1186/s13046-020-01547-5)
Supplement: Supplementary file 1 — Additional file 1 Fig. S1. Construction of MDA-MB-231 cell line with stable overexpression of CLDN6. Fig. S2. Western blotting indicating that CLDN6 overexpression downregulates HIF-1α target gene expression. Fig. S3. The correlation between CLDN6 and EMT factors. Fig. S4. CLDN6 inhibits HIF-1α deSUMOylation by down-regulating SENP1. Fig. S5. CLDN6 down-regulates SENP1 expression by blocking the nuclear translocation of β-catenin. Fig. S6. CLDN6 does not affect the expression of HIF-2α. Table S1. Clinicopathological correlation of CLDN6 expression in human breast cancers. Table S2. Clinicopathological correlation of SENP1 expression in human breast cancers. Table S3. Clinicopathological correlation of HIF-1α expression in human breast cancers. [file 13046_2020_1547_MOESM1_ESM.docx]

**SUPPLEMENTAL MATERIAL**

**A SUMOylation-dependent HIF-1α/CLDN6 negative feedback mechanism mitigates hypoxia-induced breast cancer metastasis**

Yiyang Jia^1^, Yantong Guo^1^, Qiu Jin^1^, Huinan Qu^1^, Da Qi^1^, Peiye Song^1^, Xiaoli Zhang^1^, Xinqi Wang^1^, Wenhong Xu^1^, Yuan Dong^1^, Yingying Liang^1^, Chengshi Quan^2^

1 The Key Laboratory of Pathobiology, Ministry of Education, College of Basic Medical Sciences, Jilin University, 126 Xinmin Avenue, Changchun, Jilin, 310021, People's Republic of China.

2 The Key Laboratory of Pathobiology, Ministry of Education, College of Basic Medical Sciences, Jilin University, 126 Xinmin Avenue, Changchun, Jilin, 310021, People's Republic of China. quancs@jlu.edu.cn.

**Supplemental figures**


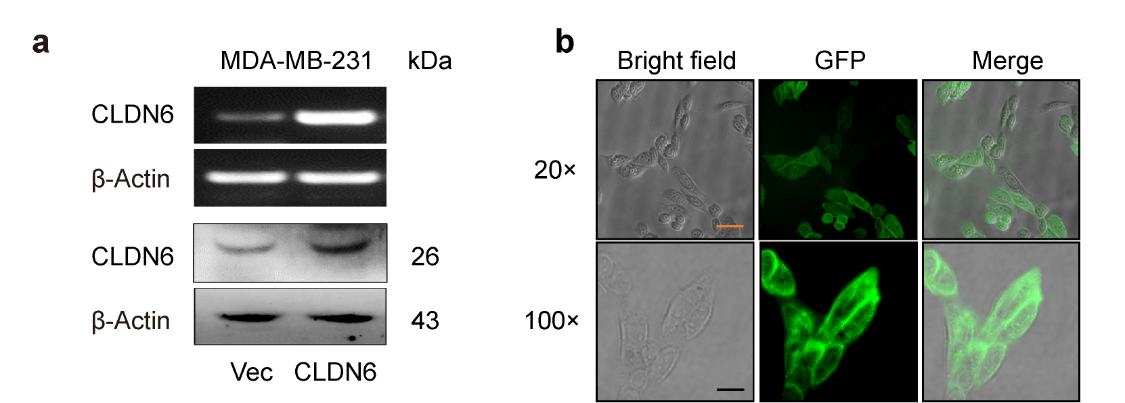


Figure.S1 Construction of MDA-MB-231 cell line with stable overexpression of CLDN6

**a** RT-PCR and western blot verified CLDN6 overexpression in CLDN6-overexpressed MDA-MB-231 cells; **b** Localization of exogenous CLDN6 in MDA-MB-231 cells by fluorescence microscopy.


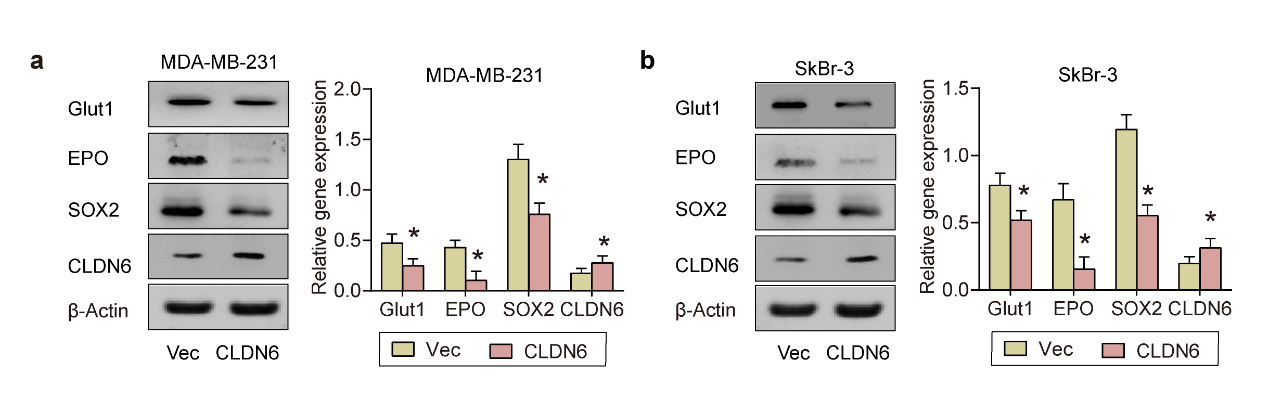


Figure.S2 Western blotting indicating that CLDN6 overexpression downregulates HIF-1α target gene expression. a Detection of Glut1, EPO and SOX2 in MDA-MB-231/vec and MDA-MB-231/CLDN6 cells. b Detection of Glut1, EPO and SOX2 in SkBr-3/vec and SkBr-3/CLDN6 cells. * *p*<0.05


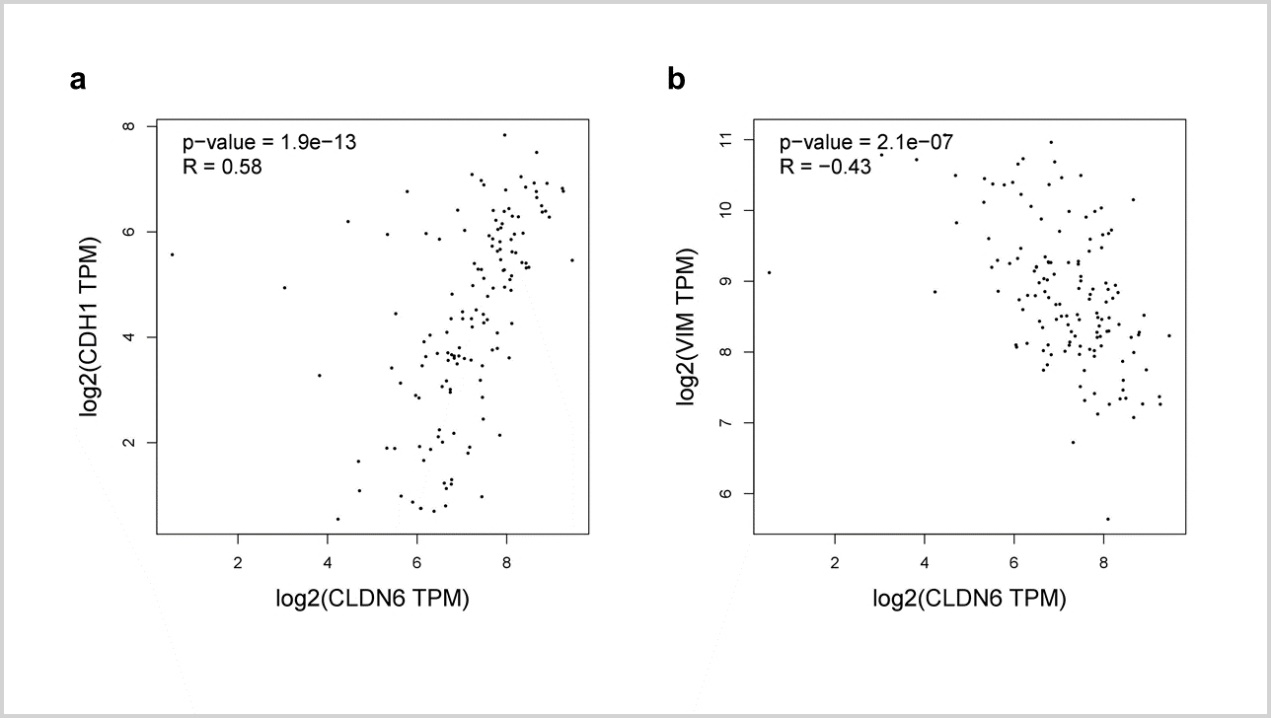


Figure.S3

The correlation between CLDN6 and EMT factors. **a** The correlation between CLDN6 and E-cadherin; **b** The correlation between CLDN6 and Vimentin.


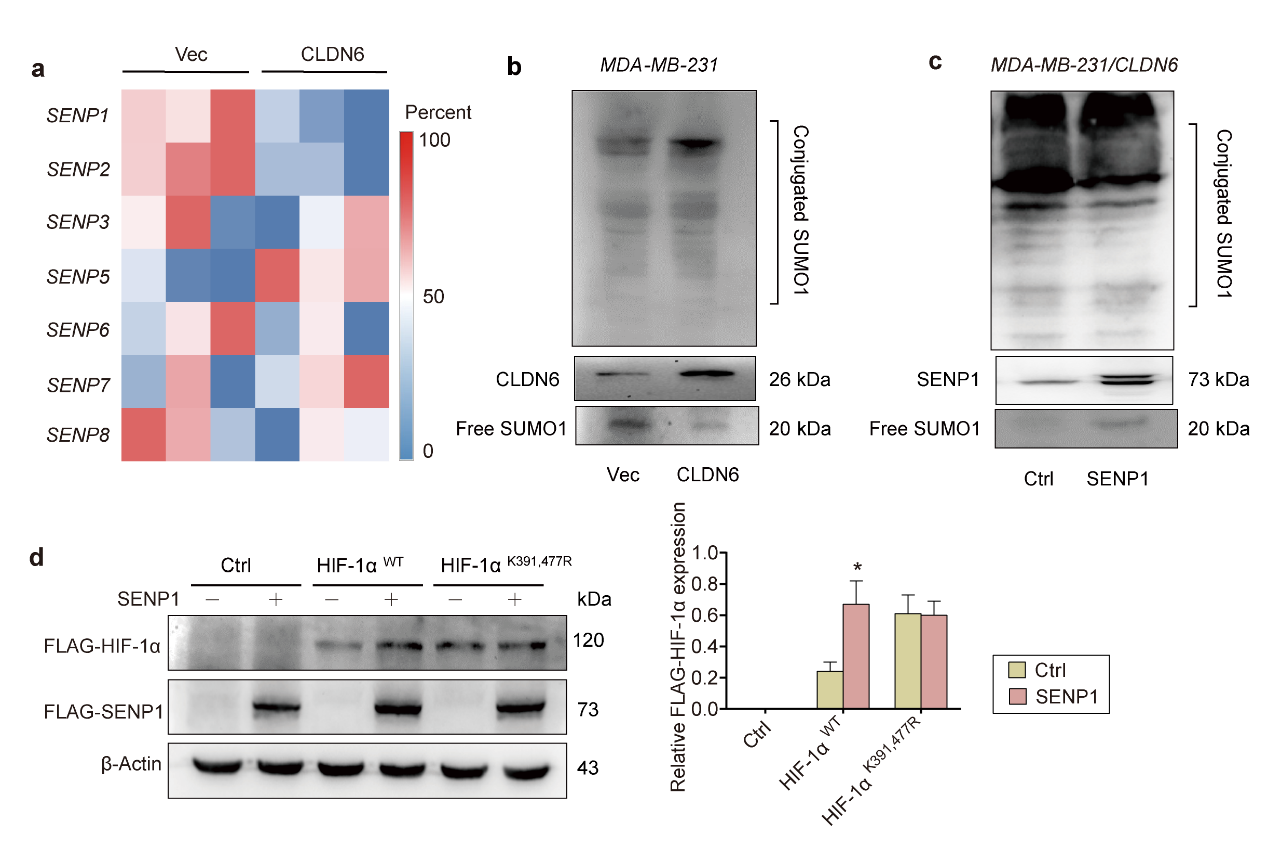


Figure.S4

CLDN6 inhibits HIF-1α deSUMOylation by down-regulating SENP1. **a** Heatmap of SENPs expression in MDA-MB-231/Vec and MDA-MB-231/CLDN6 cells; **b** Comparison of global SUMOification levels between MDA-MB-231/Vec and MDA-MB-231/CLDN6 cells; **c** Comparison of global SUMOification levels between and MDA-MB-231/CLDN6 and SENP1-OE MDA-MB-231/CLDN6 cells; **d** WB analysis indicates that SENP1 up-regulated the WT but not MUT HIF-1α expression.


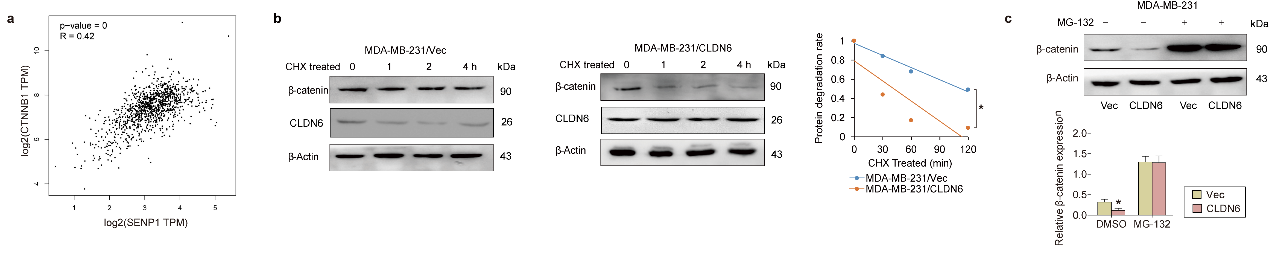


Figure.S5

CLDN6 down-regulates SENP1 expression by blocking the nuclear translocation of β-catenin. **a** The correlation between CLDN6 and β-catenin; **b** Evaluating the degradation rate of β-catenin by treating cells with CHX for 0, 1, 2 or 4 hours; **c** MDA-MB-231/Vec and MDA-MB-231/CLDN6 cells were treated with to MG-132 assess whether β-catenin is degraded by ubiquitin proteasome pathway.


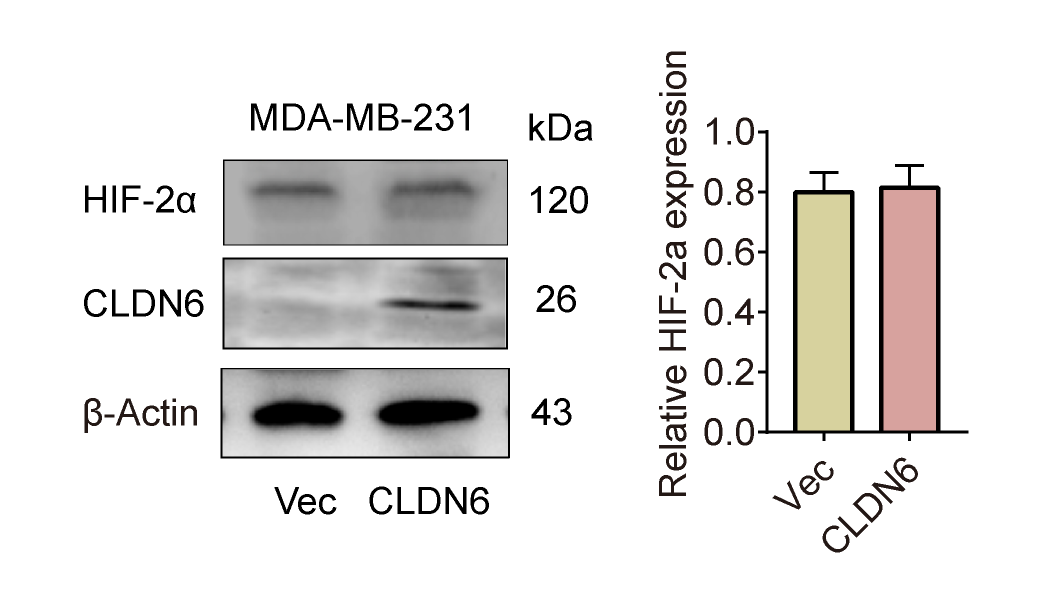


Figure.S6

CLDN6 does not affect the expression of HIF-2α.

**Table S1**

**Clinicopathological correlation of CLDN6 expression in human breast cancers**

| Characteristics of patients | | CLDN6 Expression | | | *p* |
| --- | --- | --- | --- | --- | --- |
|  |  | Low | High | Total |  |
| Age (n=50) | ≤50 | 12 | 19 | 31 | 0.369 |
|  | ＞50 | 5 | 14 | 19 |  |
| TNM Stage (n=50) | II | 4 | 15 | 19 | 0.130 |
|  | III | 13 | 18 | 31 |  |
| Histologic Grade (n=43) | 1 | 7 | 11 | 18 | 0.847 |
|  | 2-3 | 9 | 16 | 25 |  |

**Table S2**

**Clinicopathological correlation of SENP1 expression in human breast cancers**

| Characteristics of patients | | SENP1 Expression | | | *p* |
| --- | --- | --- | --- | --- | --- |
|  |  | Low | High | Total |  |
| Age (n=50) | ≤50 | 11 | 20 | 31 | 0.923 |
|  | ＞50 | 7 | 12 | 19 |  |
| TNM Stage (n=50) | II | 10 | 9 | 19 | 0.055 |
|  | III | 8 | 23 | 31 |  |
| Histologic Grade (n=43) | 1 | 9 | 9 | 18 | 0.141 |
|  | 2-3 | 7 | 18 | 25 |  |

**Table S3**

**Clinicopathological correlation of HIF-1α expression in human breast cancers**

| Characteristics of patients | | HIF-1α Expression | | | *p* |
| --- | --- | --- | --- | --- | --- |
|  |  | Low | High | Total |  |
| Age (n=50) | ≤50 | 20 | 11 | 31 | 0.280 |
|  | ＞50 | 15 | 4 | 19 |  |
| TNM Stage (n=50) | II | 15 | 4 | 19 | 0.280 |
|  | III | 20 | 11 | 31 |  |
| Histologic Grade (n=43) | 1 | 12 | 6 | 18 | 0.856 |
|  | 2-3 | 16 | 9 | 25 |  |

**Primers**

| ***Genes*** | **Primer Sequence** |
| --- | --- |
| *CLDN6* | 5'-CTGCCCATGTGGAAGGTGAC-3' |
|  | 5'-GGTAGACCAGCAAGCCGAAC-3' |
| *HIF-1α* | 5'-TTCCAGTTACGTTCCTTCGATCA-3' |
|  | 5'-TTTGAGGACTTGCGCTTTCA-3' |
| *SENP1* | 5'-ATCAGGCAGTGAAACGTTGGAC-3' |
|  | 5'-GCAGGCTTCATTGTTTATCCCA-3' |
| *VEGFA* | 5'-AAGGAGGAGGGCAGAATCAT-3' |
|  | 5'-ATCTGCATGGTGATGTTGGA-3' |
| *EPO* | 5'-TCATCTGTGACAGCCGAGTC-3' |
|  | 5'-TTTGGTGTCTGGGACAGTGA-3' |
| *Glut1* | 5'-ACAACCAGACATGGGTCCAC-3' |
|  | 5'-TAACGAAAAGGCCCACAGAG-3' |
| *SOX2* | 5'-AAATGGGAGGGGTGCAAAAGAGGAG-3' |
|  | 5'-CAGCTGTCATTTGCTGTGGGTGATG-3' |
| *VHL* | 5'-CGTAGCGGTTGGTGACTTG-3' |
|  | 5'-CCCTGGTTTGTTCCTCTGAC-3' |
| *PHD1* | 5'-CCTGAATCAGAACTGGGACGTT-3' |
|  | 5'-CGGCCCTCAGGGAAGATC-3' |
| *PHD2* | 5'-GCTTTGTTTGCCCCAGAGTATT-3' |
|  | 5'-GAATGTCCCTCCCAATCCTTAAT-3' |
| *PHD3* | 5'-GCCAGCGGTTTACCTGATAGAT-3' |
|  | 5'-TTCCCTCGCTGTGCTCCTA-3' |
| *HK2* | 5'-CCAGTTCATTCACATCATCAG-3' |
|  | 5'-CTTACACGAGGTCACATAGC-3' |
| *18s rRNA* | 5'-CGGCGACGACCCATTCGAAC-3' |
|  | 5'-GAATCGAACCCTGATTCCCCGTC-3' |
| *CLDN6* (ChIP) | 5'-GTGGTGAAGCGGAGTCTCAA-3' |
|  | 5'-GTCTCTCCAACACGCACACT-3' |
| *SENP1* (ChIP) | 5'-CCCATTCTCTGTCACTGGAGT-3' |
|  | 5'-GGGTTTCCTCCCCTGTCAAA-3' |
| *β-Actin* | CATCCACGAAACTACCTTCAACTCC |
|  | GAGCCGCCGATCCACACG |

**Antibodies**

CLDN6 Rabbit Polyclonal antibody (Bioworlde, BS3107 )

HIF-1α Mouse monoclonal antibody (Abcam, ab1)

HIF-2α Rabbit polyclonal antibody (Abcam, ab199)

SENP1 Rabbit polyclonal antibody (Proteintech, 25349-1-AP)

SUMO1 Rabbit Polyclonal antibody (Proteintech, 10329-1-AP)

Glut1 Rabbit Polyclonal antibody (Proteintech, 21829-1-AP)

EPO Rabbit Polyclonal antibody (Proteintech, 17908-1-AP)

SOX2 Rabbit Polyclonal antibody (Cell signaling technology, 3579)

E-Cadherin Rabbit Polyclonal antibody (Cell signaling technology, 3195)

N-Cadherin Rabbit Polyclonal antibody (Cell signaling technology, 13116)

Vimentin Rabbit Polyclonal antibody (Cell signaling technology, 5741)

VHL Rabbit Polyclonal antibody (Proteintech, 16538-1-AP)

PHD1 Rabbit Polyclonal antibody (Proteintech, 12984-1-AP)

PHD2 Rabbit Polyclonal antibody (Proteintech, 20368-1-AP)

PHD3 Rabbit Polyclonal antibody (Proteintech, 18325-1-AP)

FLAG Mouse monoclonal antibody (Proteintech, 66008-1-AP)

β-Catenin Mouse monoclonal antibody (Proteintech, 66379-1-AP)

α-Tubulin Rabbit polyclonal antibody (Proteintech, 11224-1-AP)

Lamin A/C Rabbit polyclonal antibody (Affinity, AF6056)

ATP1A1 Rabbit polyclonal antibody (Proteintech, 14418-1-AP)

GAPDH Mouse Monoclonal antibody (Proteintech, 60004-1-Ig)

β-Actin Mouse monoclonal antibody (Proteintech, 60008-1-AP)

| CLDN6 | Bioworlde，USA | 1:1000 | Rabbit |
| --- | --- | --- | --- |
| HIF-1α | Abcam，USA | 1:500 | Mouse |
| HIF-2α | Abcam，USA | 1:1000 | Rabbit |
| SENP1 | Proteintech，CN | 1:1000 | Rabbit |
| SUMO1 | Proteintech，CN | 1:1000 | Rabbit |
| Glut1 | Proteintech，CN | 1:1000 | Rabbit |
| EPO | Proteintech，CN | 1:1000 | Rabbit |
| SOX2 | Cell signaling, USA | 1:1000 | Rabbit |
| E-Cadherin | Cell signaling, USA | 1:1000 | Rabbit |
| N-Cadherin | Cell signaling, USA | 1:1000 | Rabbit |
| Vimentin | Cell signaling, USA | 1:2000 | Rabbit |
| VHL | Proteintech, CN | 1:1000 | Rabbit |
| PHD1 | Proteintech, CN | 1:1000 | Rabbit |
| PHD2 | Proteintech, CN | 1:1000 | Rabbit |
| PHD3 | Proteintech, CN | 1:1000 | Rabbit |
| FLAG | Proteintech, CN | 1:2000 | Mouse |
| β-Catenin | Proteintech, CN | 1:2000 | Mouse |
| α-Tubulin | Abcam, USA | 1:5000 | Rabbit |
| Lamin A/C | Affinity, CN | 1:2000 | Mouse |
| ATP1A1 | Proteintech, CN | 1:1000 | Rabbit |
| GAPDH | Proteintech, CN | 1:5000 | Mouse |
| β-Actin | Proteintech，CN | 1:10000 | Mouse |
